# A Dynamic Shelter Location and Victim Resettlement Model Considering Equitable Waiting Costs

**DOI:** 10.3390/ijerph17020471

**Published:** 2020-01-10

**Authors:** Donghai Wang, Menghao Xi, Yingzhen Chen

**Affiliations:** 1 School of Economics and Management, Beihang University, Beijing 100191, China; 2 Beijing Key Laboratory of Emergency Support Simulation Technologies for City Operations, Beijing 100191, China; 3 School of Emergency Management, Institute of Disaster Prevention, Beijing 065201, China; 4 Faculty of Maritime and Transportation, Ningbo University, Ningbo 315211, China

**Keywords:** shelter location, victim resettlement, waiting costs, fairness

## Abstract

Catastrophic natural disasters cause devastating damage and leave a huge number of homeless people. Waiting for resettlement in a post-disaster environment brings human suffering, which is defined by waiting cost in this paper. Taking into account waiting cost and fairness consideration simultaneously, a mixed integer linear programming model is constructed for the multiperiod location-allocation process. Two fairness indicators are incorporated to guarantee both the whole-process equity and the periodic equity. The model is implemented in the General Algebraic Modeling System (GAMS) and solved by the CPLEX solver. An illustrative example is provided to explain the model characteristics. Furthermore, a case study of the Yushu earthquake is conducted to demonstrate the applicability of the model to practical problems.

## 1. Introduction

Natural disasters have a devastating impact on the human society. According to the annual disaster statistical review by Below et al. (2018) [1], in 2017, 335 reported natural disasters affected 95.6 million people and caused the death of 9697 people worldwide. Besides, catastrophic natural disasters, such as earthquakes, hurricanes, and tsunamis, bring great property damage, destroy countless buildings, and leave hundreds of thousands or even millions of people homeless. Such disasters always have a long recovery period. In addition, secondary disasters continuously threaten people’s life and property. Thus, the relocation and temporary resettlement of victims are of critical importance for post-disaster response.

Since reconstruction after large-scale disasters is a complex and long-term process, providing temporary shelters to guarantee people’s safety and quality of life is one of the most urgent tasks of post-disaster response [2]. In most practical post-disaster resettlement response circumstances, people are allocated to centralized temporary resettlement sites with numerous homogeneous tents. There are many advantages of centralized resettlement, such as ease of ensuring security, improving resettlement efficiency, convenience for supply distribution, and so forth. However, in the early periods of disaster response, on one hand, disaster debris, broken roads, secondary disasters threat, and many other factors lead to limited available alternative resettlement sites. On the other hand, resources are often insufficient. Therefore, the victim resettlement process will be a multiperiod problem because shelters have to be erected gradually.

In the resettlement process, victims wait for arrangements due to limited shelter capacity and transportation capacity. The lack of access to shelter capacity or transportation capacity leads to some people becoming homeless or living in insecure dwellings temporarily, which results in human suffering reflected in physical danger and psychological stress. Osuna (1985) [3] proposes that people’s psychological stress accumulates along with the increase of waiting time. Hu et al. (2014) [4] introduce the idea into a post-disaster victim resettlement problem and present psychological cost functions to represent human suffering. From the perspective of humanitarian logistics, alleviating the suffering and guaranteeing the mental/physical health of victims is one of the most important purposes in disaster operations management. In this paper, we name the cost of waiting for resettlement waiting cost, and we consider victims’ waiting costs in a post-disaster shelter location and victim resettlement problem in a multiperiod location-allocation framework based on the thoughts of Osuna (1985) [3] and Hu et al. (2014) [4]. In addition, we take minimizing victims’ waiting costs as one of the optimization objectives instead of traditional monetary cost objectives in order to comply with the humanitarian goal of disaster operations. Furthermore, another humanitarian consideration, the fairness principle, is the other optimization objective that we focus on.

A series of guidelines are presented by Batra et al. (2005) [5], among which fairness is one of the key points. According to the document, “*resettlement must occur in a just and equitable manner*”, and “*all persons, groups and communities have the right to suitable resettlement which includes the right to alternative land or housing, which is safe, secure, accessible, affordable and habitable*”. It can be seen that fairness consideration is important and should be included in post-disaster victim resettlement decisions. Some existing studies consider fairness principles in disaster relief logistics (see, for example, [6,7,8,9]). However, resettlement planning considering fairness is still limited. In location-allocation problems, the principle of fairness requires great attention.

Traditional disaster location-allocation studies aim to reduce operation costs or transportation times, ignoring human suffering. Our research focuses on two perspectives: (1) human suffering from the perspective of waiting cost; (2) fairness. We introduce the idea of minimizing victims’ physical and mental suffering by characterizing a victim’s waiting cost as a function of waiting time. The objective guarantees fewer victims waiting for longer time periods to be allocated, which improves resettlement effectiveness. Furthermore, we consider fairness in a location-allocation problem. Fairness is incorporated from two aspects. Firstly, we adopt a min–max method to minimize the maximum unit waiting cost gap between two different disaster areas. This objective makes sure that victims in different areas have a fair priority of allocation and it is not preferred that victims in a particular area wait for a much longer time to be allocated and incur more human suffering. Secondly, we introduce periodic equity consideration, which is achieved by a constraint of a required allocation ratio (or service level). This consideration can guarantee that victims in different areas feel equally treated in each period. 

Based on the above considerations, this study simultaneously addresses disaster human suffering (which is expressed by waiting cost) and fairness principles in a dynamic multiperiod location-allocation (DMPLA) problem. The main contributions of this paper are summarized as follows:

- We simultaneously consider waiting cost and fairness in the DMPLA problem.
- We consider fairness from two aspects. Firstly, we consider the unit waiting cost gap between two different disaster areas over the whole victim resettlement process. Secondly, we set an allocation percentage (or service level) threshold in each time period.
- We formulate a mixed integer linear programming model and a numerical example is investigated to explain the model. Furthermore, a case study is conducted to demonstrate the applicability of the DMPLA model to practical problems.

The remainder of this paper is organized as follows. Section 2 reviews the related literature. Section 3 provides the description of our problem. Section 4 defines waiting cost and fairness, and then formulates the DMPLA model. Section 5 designs an illustrative example to explain the model. Section 6 provides a case study and result discussions. Section 7 summarizes the findings of the paper and provides further research directions.

## 2. Literature Review 

Our model combines several aspects, namely shelters, temporary resettlement, human suffering, and fairness in a multiperiod location-allocation framework. In this section, we review the related literature mainly focusing on these aspects.

### 2.1. Shelter Location and Victim Resettlement

Shelters have different functions in different phases of disasters. Félix et al. (2013) [2] provide a subdivision of shelters based on victims’ residence time. They define a temporary shelter as a place “*used for an expected short stay, ideally no more than a few weeks after the disaster, this may be a tent, a public mass shelter, etc*”. This definition is closest to ours in this study. 

Recently, most shelter location optimization models in disaster operations management have combined the process of shelter location with evacuation or victim allocation. These location-evacuation or location-allocation models generally seek to pick shelters from a given set of alternative locations and provide transportation plans to minimize the total costs or evacuation time [10]. Sherali et al. (1991) [11] propose a location-allocation model to select shelters from available locations and provide an evacuation plan to minimize the total congestion-related evacuation time. Kongsomsaksakul et al. (2005) [12] use a Stackelberg game to express the shelter location and victim evacuation problem. The authority acts as the leader who determines shelter locations to minimize the total evacuation time, and the evacuees act as the follower choosing the route to reach a shelter as quickly as possible. Adopting similar objectives, Li et al. (2012) [13] develop a scenario-based bilevel optimization model for shelter location and evacuation planning. Kulshrestha et al. (2011) [14] propose a robust method for determining locations and capacities of public shelters during evacuation under demand uncertainty, which contributes to minimize the total cost to establish and operate the shelters. Rawls et al. (2012) [15] present a dynamic allocation model to optimize pre-disaster mitigation strategy for shelter readiness, which aims to minimize the total costs including location and inventory-related costs. The above research presents models mainly focusing on monetary cost or travel time, rather than directly reflecting the humanism perspective.

Different from traditional cost-oriented or time-oriented objectives, Farahani et al. (2018) [16] propose a mixed integer linear programming model which combines location decisions with the max-flow problem. The objective is to select locations which maximize the number of dispatched people. Hu et al. (2014) [4] propose that the panic of victims increases with the increase in waiting time for evacuation. They formulate a multiperiod location-evacuation model considering victim panic and panic spread to minimize victims’ panic-induced psychological costs and other monetary costs. Based on their thought, this study simultaneously considers people’s waiting costs for resettlement and equity from the aspects of behavioral and social science.

### 2.2. Human Suffering

In recent years, the consideration of human suffering in humanitarian logistics has attracted increasing attention. Currently, there are two main streams considering the quantification of human suffering in disaster operations management. 

One stream of studies quantifies the human suffering of people as a deprivation cost, which is defined by Holguín-Veras et al. (2013) [17] as: “*…the economic value of the human suffering caused by a lack of access to a good or service*”. They postulate that a deprivation cost function is likely to be a monotonic, nonlinear, and convex function of time. However, deprivation cost functions are almost always evaluated for one specific kind of resource and are merely applied to disaster relief logistics, which are not suitable for quantifying human suffering caused by waiting for evacuation. 

The other stream of research considers human suffering from the perspective of psychological cost. Osuna (1985) [3] proposes that people’s psychological stress accumulates while waiting, which can be characterized as an increasing function of waiting time. Hu et al. (2013) [18] introduce this thought into disaster operations. They characterize the psychological cost as an increasing function of the waiting time for medical treatment and debris removal during a debris reverse logistic process. In their another research [4], a similar thought is applied to describe victims’ panic degree. They formulate a multiperiod location-evacuation model considering victim panic and panic spread. Their research provides a theoretical basis for the study of shelter location and victim resettlement considering human suffering.

### 2.3. Fairness

In addition to efficiency and effectiveness, fairness is another important consideration of the management and decision sciences. Marsh et al. (1994) [19] list 20 measures of equity in location models, including range, variance, mean deviations, Gini index, and others. These equity metrics are widely used in general location problems [20,21,22,23,24]. 

An increasing number of scholars include equity consideration in disaster operations models. However, most fairness-related research focuses on relief logistics. Most of these studies adopt fairness as an objective function to optimize. Lin et al. (2011) [6] propose a multiobjective model in post-disaster relief operations. One of the objectives is to guarantee equity by minimizing the maximum gap of the satisfaction rate between two arbitrary different demand nodes. Manopiniwes et al. (2017) [7] consider equity by using a *p*-center approach to minimize the maximum response time between facilities and disaster sites in a relief supply chain. Hu et al. (2016) [8] consider fairness by maximizing the minimal satisfaction rate of demand points in a disaster relief allocation problem. Huang et al. (2012) [9] introduce three equity metrics in a vehicle routing and supply allocation problem, two of which are deviation-type equity metrics (range and standard deviation), and they are adopted as optimization objectives. The third metric compromises fairness and efficiency by considering time index, which is adopted for evaluation purposes. Few studies consider fairness in shelter location and victim allocation problems. We express equity through both objective optimization and hard constraints (service level) in a multiperiod location-allocation framework in order to enrich existing research.

From the literature review, we observe that disaster location-evacuation or location-allocation models considering quantified human suffering are still limited, especially for research that pays attention to fairness and equity. To enrich and supplement the literature in the disaster operations area, this study addresses a post-disaster shelter location and victim resettlement problem, which simultaneously considers human suffering and equity in a multiperiod location-allocation framework.

## 3. Problem Description

**Figure 1:**
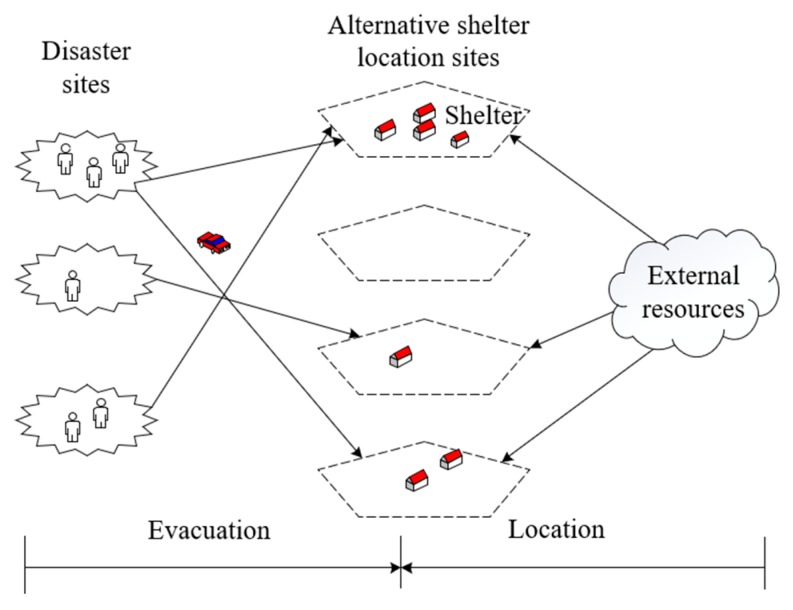
Post-disaster location-evacuation network.

Our problem makes two sets of decisions, namely, where to locate shelters in each period and how many victims to allocate from one demand point to a shelter location in each period (see Figure 1). Further details and assumptions of the mentioned two aspects of decisions are described below.

### 3.1. Shelter Location

In the emergency network, a set of safe candidate location points are given to erect shelters. We assume that in different periods, available location sets are different. This is reasonable because some roads and location sites need to be cleaned up or repaired. So, in the early periods, maybe some location points are not available. In disaster response, shelters are always built up by standardized and easily erected tents or board rooms. Thus, we assume that all newly built shelters have the same capacity. We further assume that each location point can erect only one shelter, which contains numerous homogeneous tents. A limited number of shelters can be built during each time period. The shelters’ establishment time is ignored because they are made up of easy-to-build tents. The local transportation and setup process of tents does not conflict with the transportation process of victims. From another perspective, the shelter number constraint can also explain this setting. Even if the shelter establishment is assumed to be time-consuming, we only need to set the number that can be established in the current period as the constraint.

In summary, the shelter location decision solves the problem of picking which several points to use for building shelters from a set of candidates during each time period.

### 3.2. Victim Allocation

After a disaster, victims from a set of disaster sites need to be relocated. Affected regions can be viewed as different demand points, and the demand is unallocated victims of each region. In this paper, the victims waiting for allocation refer to people who have no ability to self-evacuate and need to be uniformly allocated by the government. Based on the classification of transportation vehicles for evacuation in Russo et al. (2011) [25], we assume the victims are transported by motorized vehicles (like buses) in batch, and some people have to wait for allocation, which increases their waiting cost. In a good allocation plan, fewer people wait for a longer time to be allocated. Besides, fairness is a key consideration in disaster response, which has been widely researched in disaster relief logistics. 

Based on the above description, our study aims to reduce people's waiting costs while ensuring decision equity. The formulation of the DMPLA model and the definitions of waiting cost and equity will be introduced in Section 4.

## 4. Formulation

The list of notations is as follows.

(1) Indices

*i*: Index for demand point, i=1,2,…,I

*j*: Index for potential shelter location site

*t*: Index for time period, t=1,2,…,T

Sets:

$J^{t}$: Set of all potential shelter location sites for period t. Without loss of generality, we assume that $J^{1}\subseteq J^{2}\subseteq \ldots \subseteq J^{T}$

(2) Parameters

$A_{i}$: Initial demand at demand point i, i=1,2,…,I

C: Capacity per shelter

$S^{t}$: Transportation capacity in period t, t=1,2,…,T

$B^{t}$: The budget number of shelters for period t, t=1,2,…,T

$q^{t}$: Service level requirement for period t, t=1,2,…,T

$d_{ij}^{t}$: Distance from demand point i to location j during period t, t=1,2,…,T, i=1,2,…,I, $j\in J^{t}$

α: Weight for equity

$w_{t}$: One unit waiting cost of each person waiting *t* periods, t=1,2,…,T−1

(3) Decision variables

$x_{ij}^{t}$: Number of people moved from demand point i to shelter j in period t, t=1,2,…,T, i=1,2,…,I, $j\in J^{t}$, $x_{ij}^{t}\geq 0$

$y_{j}^{t}$: 0-1 variable of whether to open a shelter at location j in period t*,* t=1,2,…,T, $j\in J^{t}$

$D_{i}^{t}$: Number of people waiting to be allocated from demand point i at the beginning of period t, t=2,3,…,T, i=1,2,…,I, $D_{i}^{t}\geq 0$

$R_{j}^{t}$: Remaining capacity at location j at the end of period t, t=1,2,…,T−1, $j\in J^{t}$, $R_{j}^{t}\geq 0$

WC: Total waiting cost

E: Dummy variable for equity, E≥0

### 4.1. Waiting Cost

Waiting cost is one of the optimization objectives of our model. We denote $w_{t}$ as the unit waiting cost of each person waiting *t* periods. We introduce the thought of Hu et al. (2013, 2014) [4] and set $w_{t}$ as a monotonically increasing function of waiting time, which can be linear (γt), quadratic ($\gamma t^{2}$), or exponential ($\gamma e^{t}$) , where γ is a constant.

Then, the waiting cost of people delivered during time period t (who have waited for t−1 periods) in point i can be denoted by $\sum_{j\in J^{t}}w_{t-1}x_{ij}^{t}$. For example, people who are allocated during Period 3 have already waited for 2 periods and their waiting costs equal to $\sum_{j\in J^{3}}w_{2}x_{ij}^{3}$. Thus, WC is denoted by $\sum_{t=2}^{T}\sum_{i=1}^{I}\sum_{j\in J^{t}}w_{t-1}x_{ij}^{t}$, where T denotes the last time period. 

### 4.2. Fairness

We propose two metrics of attributes to define fairness. One is waiting cost equity, which is adopted for an objective. The other is periodic service level equity, and we adopt it as a hard constraint. The two fairness attributes are defined as follows.

#### 4.2.1. Waiting Cost Equity

Waiting cost equity denotes the maximum gap of unit waiting cost between any two different demand points. As described previously, the total waiting cost of point i is denoted by $\sum_{t=2}^{T}\sum_{j\in J^{t}}w_{t-1}x_{ij}^{t}$. 

Let E be the maximum gap between any two different demand points. Then,

$$E\geq \frac{\sum_{t=2}^{T}\sum_{j\in J^{t}}w_{t-1}x_{ij}^{t}}{A_{i}}-\frac{\sum_{t=2}^{T}\sum_{j\in J^{t}}w_{t-1}x_{i^{\prime }j}^{t}}{A_{i^{\prime }}},i\neq i^{\prime },\;i\&i^{\prime }=1,2,\ldots ,I.\;$$

In the following model, we take E as the other objective to minimize. This means that the model aims to make people’s waiting cost difference between any two different demand points as small as possible, which reflects the fairness thinking. 

#### 4.2.2. Service Level Equity

Another measure to guarantee fairness is that we set a service level constraint in our model. We define the service level $q^{t}$ as the required allocation proportion during period t. Then,

$$\left\{ \begin{array}{ll} \sum_{j\in J^{1}}x_{ij}^{1}\geq q^{1}A_{i},\; & i=1,2,\ldots ,I,\\ \sum_{j\in J^{t}}x_{ij}^{t}\geq q^{t}D_{i}^{t},\; & t=2,3,\ldots ,T,\;i=1,2,\ldots ,I. \end{array}\right.$$

This service level constraint is a complement for the first equity indicator. The waiting cost equity embodies fairness on the aspect of the entire resettlement process, while the service level constraint requires that people in each demand point be allocated at a rate not less than the periodic requirement service level. 

### 4.3. Mathematical Model

Based on the above descriptions, now we present the DMPLA model.

**Objective function**

The DMPLA model minimizes the weighted sum of the total waiting costs and equity, that is:

(1) Min WC+αE

**Constraints**

The DMPLA model requires the following set of constraints to be satisfied:

(2) $$\sum_{t=1}^{T}\sum_{j\in J^{t}}x_{ij}^{t}=A_{i},\;i=1,2,\ldots ,I;$$

(3) $$\sum_{j\in J^{t}}y_{j}^{t}\leq B^{t},\;t=1,2,\ldots ,T;$$

(4) $$\sum_{t=1}^{T}y_{j}^{t}\leq 1,\;j\in J^{1};$$

(5) $$\sum_{t^{\prime }=t}^{T}y_{j}^{t^{\prime }}\leq 1,\;t=2,3,\ldots ,T-1,\;j\in J^{t}\backslash J^{t-1};$$

(6) $$D_{i}^{2}=A_{i}-\sum_{j\in J^{1}}x_{ij}^{1},\;i=1,2,\ldots ,I;$$

(7) $$D_{i}^{t+1}=D_{i}^{t}-\sum_{j\in J^{t}}x_{ij}^{t},\;t=2,3,\ldots ,T-1,\;i=1,2,\ldots ,I;$$

(8) $$\sum_{i=1}^{I}x_{ij}^{1}\leq Cy_{j}^{1},\;j\in J^{1};$$

(9) $$\sum_{i=1}^{I}x_{ij}^{t}\leq R_{j}^{t-1}+Cy_{j}^{t},\;t=2,3,\ldots ,T,\;j\in J^{t-1};$$

(10) $$\sum_{i=1}^{I}x_{ij}^{t}\leq Cy_{j}^{t},\;t=2,3,\ldots ,T,\;j\in J^{t}\backslash J^{t-1};$$

(11) $$R_{j}^{1}=Cy_{j}^{1}-\sum_{i=1}^{I}x_{ij}^{1},\;j\in J^{1};$$

(12) $$R_{j}^{t}=R_{j}^{t-1}+Cy_{j}^{t}-\sum_{i=1}^{I}x_{ij}^{t},\;t=2,3,\ldots ,T-1,\;j\in J^{t-1};$$

(13) $$R_{j}^{t}=Cy_{j}^{t}-\sum_{i=1}^{I}x_{ij}^{t},\;t=2,3,\ldots ,T-1,\;j\in J^{t}\backslash J^{t-1};$$

(14) $$\sum_{i=1}^{I}\sum_{j\in J^{t}}d_{ij}^{t}x_{ij}^{t}\leq S^{t},\;t=1,2,\ldots ,T;$$

(15) $$E\geq \frac{\sum_{t=2}^{T}\sum_{j\in J^{t}}w_{t-1}x_{ij}^{t}}{A_{i}}-\frac{\sum_{t=2}^{T}\sum_{j\in J^{t}}w_{t-1}x_{i^{\prime }j}^{t}}{A_{i^{\prime }}},\;i\neq i^{\prime },\;i\&i^{\prime }=1,2,\ldots ,I;$$

(16) $$\sum_{j\in J^{1}}x_{ij}^{1}\geq q^{1}A_{i},\;i=1,2,\ldots ,I;$$

(17) $$\sum_{j\in J^{t}}x_{ij}^{t}\geq q^{t}D_{i}^{t},\;t=2,3,\ldots ,T,\;i=1,2,\ldots ,I.$$

Constraint (2) guarantees that all the people are allocated by the prescribed last time period. 

Constraint (3) ensures that a limited number of shelters can be built in each period.

Constraints (4) and (5) together express that one candidate location point is either chosen or not. 

Constraints (6) and (7) show that the number of people waiting for resettlement at each point at the beginning of each time period equals that of the previous period minus the number of people who are allocated during the previous period. 

Constraints (8)–(10) together guarantee that the number of people allocated to one location site does not exceed its available capacity. In Constraint (8), the available capacity of point j equals the unit capacity C if point j is chosen at the beginning of the first period, else it equals zero. In Constraint (9), point j is not a newly added point during period t, t=2,3,…,T. Thus, the available capacity of point j has two situations. If it has been chosen in any previous period,$y_{j}^{t}=0$, referring to Constraints (4) and (5). In this situation, the available capacity of point j during period t equals the remaining capacity of point j at the end of period *t* − 1, which is denoted by $R_{j}^{t-1}$. If it has not been chosen in any previous period, then $R_{j}^{t-1}=0$. The available capacity of point j equals $Cy_{j}^{t}$, which depends on whether the point is chosen in period t. In Constraint (10), point j is a newly added point during period t. Thus, the available capacity of point j depends on whether it is chosen because there is no remaining capacity. 

Constraints (11)–(13) are balance constraints. They express that the remaining capacity at shelter location j at the end of period t equals the available capacity at the beginning of the period minus the number of people who are allocated to point j during period t. Three situations are expressed in a similar way to Constraints (8)–(10).

Constraint (14) ensures the periodic transportation capacity limit. Here we take the product sum of allocated number from demand point i to location point j and the distance between them during period t as the periodic transportation amount. This capacity depends on the vehicle capacity and daily service frequency of a transport line. For example, if the vehicle capacity is 30, and the length and service frequency of a transport line are 10 km and 4 runs/day, respectively, then the daily transportation capacity of this line is 1200 passengers*km.

Constraints (15)–(17) have been described previously referring to Section 4.2. 

The formulas (1)–(17) together represent the DMPLA model, which is a mixed integer linear program. 

The disaster management process is a complex project which is supported by a system of models, such as demand models, path choice models, refuge area simulation models, and so forth [26]. The DMPLA model is a part of the overall decision system. For example, the outputs of the DMPLA model provides a basis for further decisions, including the vehicle assignment, supply transportation, and so on. Conversely, the implementation of the DMPLA model also needs the support of other optimization components. For example, the pre-disaster relief reserve impacts the shelter number constraint, user behavior consideration in demand models contributes to give more realistic periodic demand, and so forth.

## 5. Illustrative Example

We now provide an illustrative example to explain the DMPLA model. We consider a small-scale example with four demand points and five candidate points for location. A duration of five time periods is considered. For simplicity, we assume that the distance between any two points is constant in the illustrative example.

In the study of Hu et al. (2014) [4], the authors conclude that the quadratic mode is an appropriate description of psychological cost through questionnaire and interviews. Based on this, we adopt a quadratic monotonically increasing function to express unit waiting cost in this section and the following case study. The expression of $w_{t}$ is presented as $\gamma t^{2}$, where *γ* is a constant. Based on the above settings, we conduct two groups of experiments to show the features of the DMPLA model. The model parameters and their values for this section refer to Table 1 and Table 2. All models are implemented in GAMS and solved using CPLEX as the optimizer.

### 5.1. Fairness Interpretation

**Figure 2:**
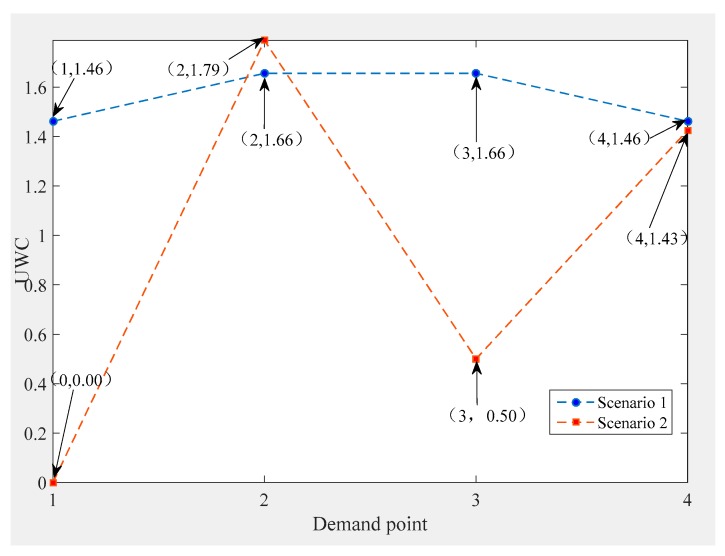
Unit waiting cost (UWC) of two scenarios.

We consider two scenarios for the DMPLA model. In Scenario 1, we use the basic parameter settings. In Scenario 2, we do not consider fairness and set parameter α and $q^{t}$ to 0. Obviously, a more equitable solution is obtained in Scenario 1 (see Figure 2). Meanwhile, the total waiting cost in Scenario 1 also increases compared to Scenario 2. The increasing degree depends on the value of *α*.

The fairness consideration leads to a trade-off between victims’ waiting cost and decision equity. This makes sense in practical decisions. The objective of the DMPLA model is to obtain relatively good arrangements for everyone instead of some people getting extremely good arrangements while some people get extremely bad arrangements. For example, if people face a high risk of acquiring serious physical or mental illness for waiting more than five days, we prefer to try to keep everyone waiting no more than five days even though this may lead to an increase of waiting time for some people who could have waited for a very short time. Of course, in practical decisions, we will avoid sacrificing too much waiting cost while ensuring fairness through setting appropriate fairness weights. Further, a sensitivity analysis of α will be conducted in Section 6.

### 5.2. Waiting Cost Verification 

In this section, we compare the DMPLA objective to a traditional cost-oriented objective. In our problem, monetary cost is represented as follows:

$$MC=cb*\sum_{t=1}^{T}\sum_{j\in J^{t}}y_{j}^{t}+ct*\sum_{t=1}^{T}\sum_{i=1}^{I}\sum_{j\in J^{t}}d_{ij}^{t}x_{ij}^{t},$$

where cb denotes the unit shelter building cost, and ct denotes the transportation cost for delivering one victim per kilometer. 

We replace WC in the DMPLA model with MC. Correspondingly, the equity objective changes accordingly. We name the new model the P1 model. In the P1 model, we take the gap of unmet demand between different regions as a measure of equity objective, which is denoted as E1. Then, the model is presented as follows.

P1 model:

 min MC+αE1

 s.t. Constraints (2)–(14);

    Constraints (16)–(17);

$$E1\geq \frac{\sum_{t=1}^{T}\left(D_{i}^{t}-\sum_{j\in J^{t}}x_{ij}^{t}\right)}{A_{i}}-\frac{\sum_{t=1}^{T}\left(D_{i^{\prime }}^{t}-\sum_{j\in J^{t}}x_{i^{\prime }j}^{t}\right)}{A_{i^{\prime }}},i\neq i^{\prime },\;i\&i^{\prime }=1,2,\ldots ,I.\;$$

**Figure 3:**
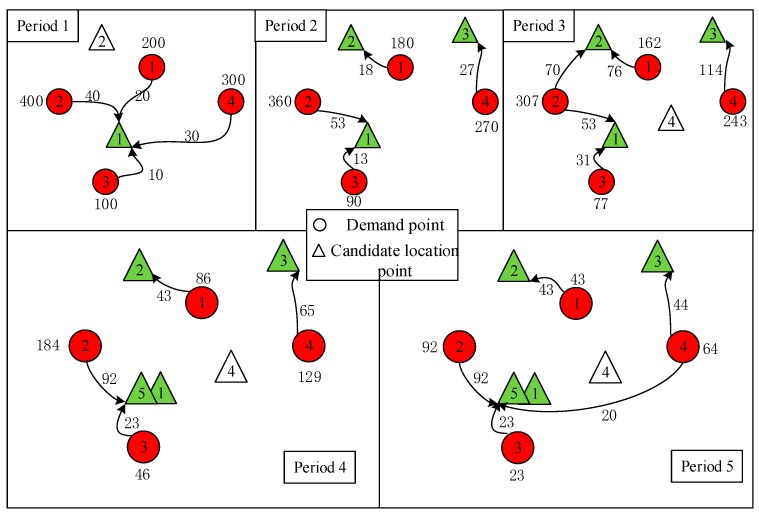
Results of P1 model.

**Figure 4:**
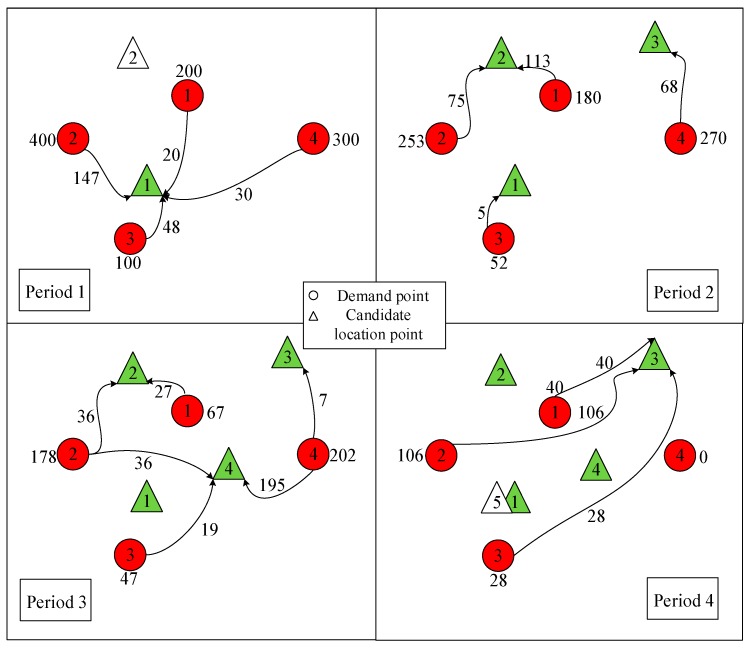
Results of DMPLA model.

**Figure 5:**
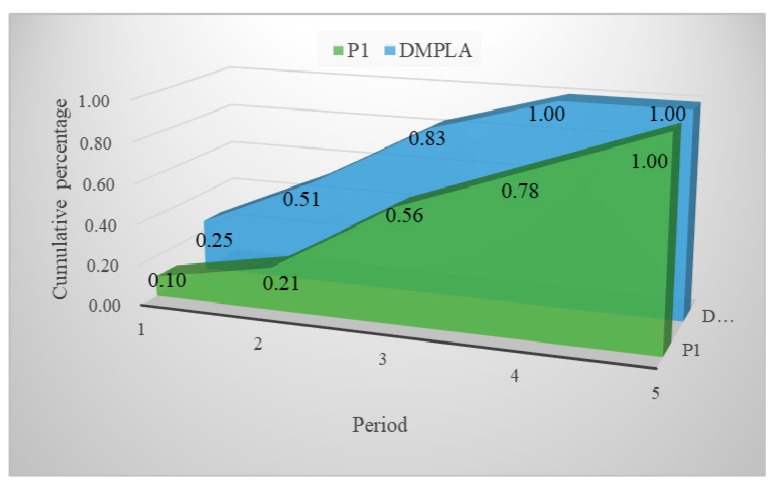
Cumulative allocation percentage of the two models.

The results of P1 and DMPLA are shown in Figure 3 and Figure 4. Furthermore, a cumulative allocation percentage of the models is presented in Figure 5.

We see that in the P1 model, 455 people, who occupy a percentage of 44.5%, are delivered during the last two periods, while in the DMPLA model, 83% people have been allocated by the end of Period 3. All the people are allocated by the end of Period 4. Consequently, the corresponding waiting costs of the P1 model and the DMPLA model are 3526 and 1559, respectively. Meanwhile, the total monetary costs of the P1 model and the DMPLA model are 1,626,000 *yuan* and 1,637,700 *yuan*, respectively. Compared to the P1 model, the DMPLA model reduces the waiting costs by 55.79% with a slight increase of monetary costs by 0.72%. The differences are because the P1 model aims to find the optimal solution which minimizes the total monetary costs throughout the whole resettlement process. Thus, people are more likely to be allocated until the good candidate points (i.e., points covering more demand, for example, candidate point 5) appear, which leads to the increasing of waiting costs. In comparison, the DMPLA model aims to reduce people’s waiting costs within a monetary budget acceptable to decision makers. 

In summary, the DMPLA model is beneficial to reduce people’s waiting time for allocation by minimizing waiting costs. Even though monetary costs increase by a certain extent compared to traditional cost-oriented models, it is controllable under the constraints of building capacity and transportation capacity. In addition, the DMPLA model provides an equitable decision.

## 6. Case Study

### 6.1. Case Background and Data Estimation

On April 14, 2010, a 7.1-magnitude earthquake struck the Yushu prefecture in the Qinghai Province in China. According to the official toll, the earthquake affected an area of 30,000 square kilometers, killed 2698 people (including 270 missed), and injured 12,135 people. Furthermore, the disaster destroyed at least 85% of houses in Yushu County and left about 100,000 victims homeless and waiting for relocation. In addition to deaths, physical injuries, and economic losses, the disaster brought psychological suffering to victims. The earthquake caused prevalence rates of post-traumatic stress disorder, anxiety, and depression up to 33.7%, 43.8%, and 38.6% among survivors, respectively [27]. The government dispatched emergency tents throughout the country and almost all victims were relocated to resettlement sites within five days after the earthquake; in the case study, we assumed a seven-day duration, and each day denotes one period.

We took the Yushu earthquake as the background of case studies to verify the applicability of the DMPLA model. The data estimation is presented as follows.

#### 6.1.1. Initial Demand of Each Disaster Site

**Figure 6:**
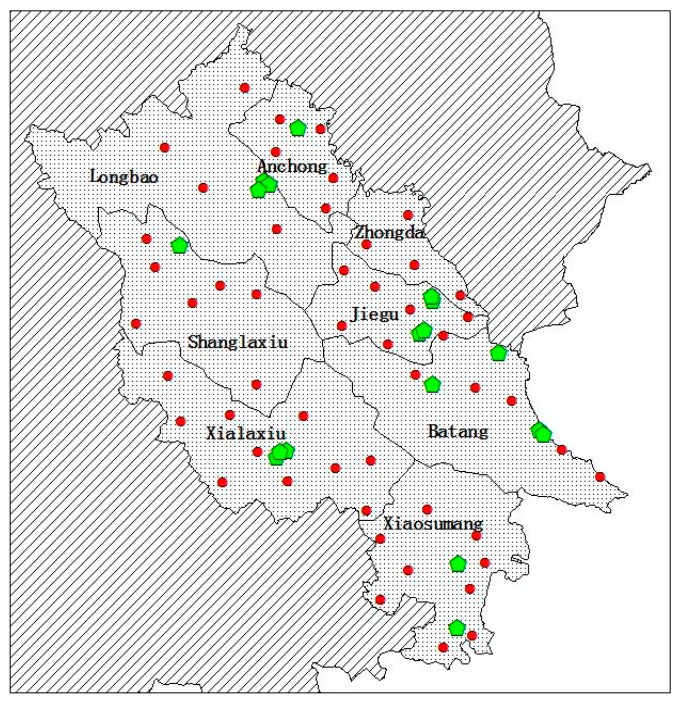
Geographic locations of disaster sites and part of candidate location points in Yushu County (dots denote disaster sites and pentagons denote location points).

As is shown in Figure 6, 8 towns of Yushu County were affected in the earthquake, and 51 villages in the 8 towns were selected as demand points. Based on population density and geographic structure, we simulated 60 candidate shelter location points. The candidate location points were opened gradually. We assumed that each shelter location point had the same capacity of 2000.

According to the data of China’s Sixth Population Census, we obtained the proportion of population in each township. Then, we estimated the initial population to be resettled in each disaster site in proportion. 

#### 6.1.2. Initial Distance between a Demand Point and a Candidate Shelter Location Point

We estimated the initial driving distance between a demand point and a candidate shelter location point under the support of a Geographic Information System. 

#### 6.1.3. Monetary Costs

We followed the estimation of unit transportation cost and building cost in an existing research [4]. The unit transportation cost was 2 *yuan* and the unit cost of allocating one person was 1600 *yuan*; accordingly, the unit building cost for one shelter was 3,200,000 *yuan*.

#### 6.1.4. Periodic Capacities for Victim Transportation and Building Shelters 

Periodic capacities for victim transportation and building shelters were estimated by news and government reports. The periodic building capacity was determined by relief reserve, donations, and emergency purchasing. The periodic transportation capacity was determined by road conditions, available vehicles, and manpower. 

#### 6.1.5. Weight for Equity

In the base parameter settings, the equity weight was set to be 0.12*10^5^ and the magnitude of 10^5^ was determined by the total demand. The later equity weight sensitivity analysis guided the setting of equity weight.

#### 6.1.6. Periodic Service Level 

The setting of periodic service level constraints is significantly influenced by other parameter settings. Setting a fixed value for service level is not conducive to the sensitivity analysis of other parameters. Thus, the original service level requirement for each period was set to 0. A sensitivity analysis was conducted for the service level parameter later.

### 6.2. Numerical Results 

This section presents experiment designs and numerical results to demonstrate the efficacy of the DMPLA model. Table 3 summarizes the experimental scenarios, corresponding purposes, and experimental steps. For each scenario, we ran the models using Cplex solver by GAMS with a computer processor of Intel® Core™ i3-3240 CPU @ 3.40Ghz and 4.00 G installed memory (RAM).

**Figure 7:**
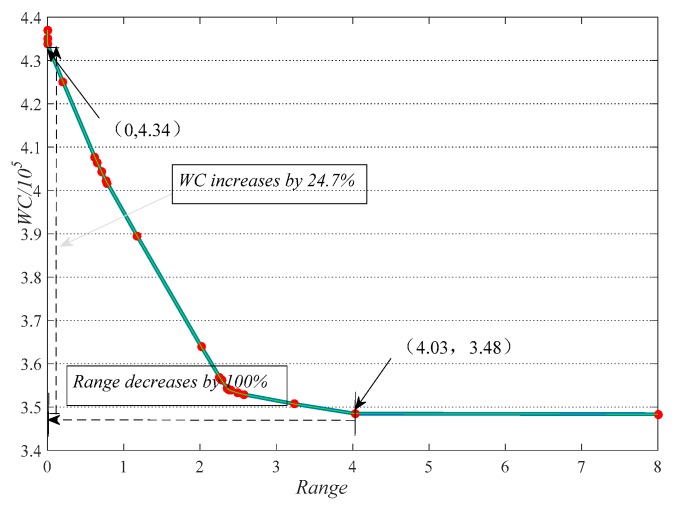
A Pareto front between waiting cost and equity (Range).

**(I)** By solving the DMPLA model with different equity weights, 101 pairs of waiting costs and equity values were achieved. The distribution of solutions and their Pareto front are presented in Figure 7. We see that in the DMPLA model, the inequity could be completely eliminated by increasing waiting costs by 24.7%. Further, we see that the growth rate of waiting cost (2.59%) was relatively slow when the value of *E* was between 2.26 and 4.03. The corresponding equity weight was between 0.01 × 10^5^ and 0.29 × 10^5^. When the equity weight was not less than 0.34 × 10^5^ , the decision reached absolute equity. 

This provides guidelines for the setting of equity weight in practical decision-making processes.

**Figure 8:**
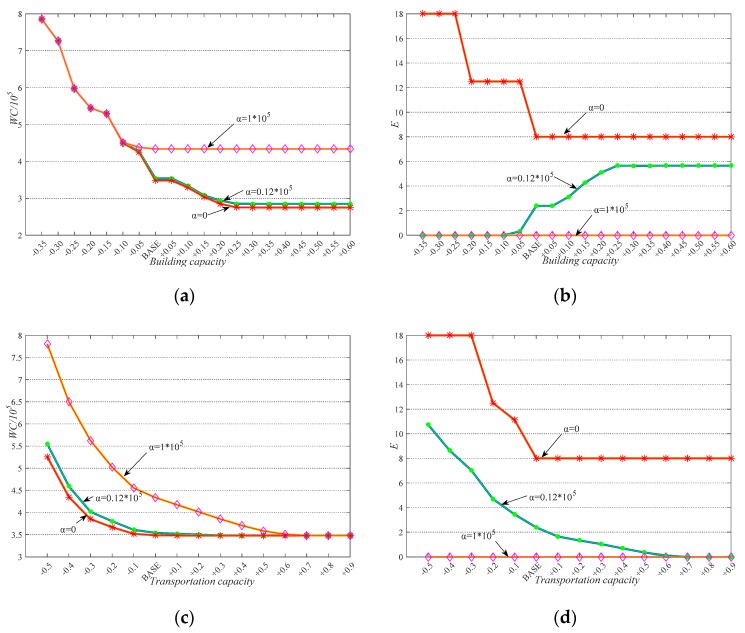
Periodic capacities sensitivity analysis: (**a**) The impact of building capacity on WC; (**b**) the impact of building capacity on E; (**c**) the impact of transportation capacity on WC; (**d**) the impact of transportation capacity on E.

**(II)** The results of experiments II and III are together presented in Figure 8. Several findings are presented as follows.

**a.** From (a) and (c), we see that the increase of building capacity and transportation capacity both contribute to the decrease of waiting cost.

**b.** From (b) and (d), focusing on the scenario that α equals to 0.12 × 10^5^, we see that the increase of transportation capacity contributes to the improvement of equity, while the increase of building capacity leads to inequity instead. This is because transportation capacity is a stronger constraint than building capacity in our problem. This is easy to understand since transportation capacity not only limits the number of people delivered but also limits the delivery distance, while building capacity mainly limits the number of allocated people. Thus, even if building capacity is improved in (b), the model objective is still not easy to be improved due to the limitation of transportation capacity. Then the objective is improved through the tradeoffs between waiting cost and equity, instead of improving waiting cost and equity simultaneously.

**c.** From (a) and (b), we see that when building capacity is in a small value interval, the different values of *α* do not impact the value of waiting cost. However, the value of equity is significantly decreased when non-zero values are set for *α* compared to the zero-value circumstance. This implies that the equity consideration does not necessarily lead to the increase in waiting cost. In addition, we see that when building capacity constraint is tight, the decision is fairer. This is because in this scenario, people have to wait a long time until enough shelters are erected. In practice, this situation is common because during the first 72 h after a disaster emergency, supplies are very limited. When the waiting cost is difficult or even impossible to decrease, the DMPLA model contributes to improving fairness. 

**Figure 9:**
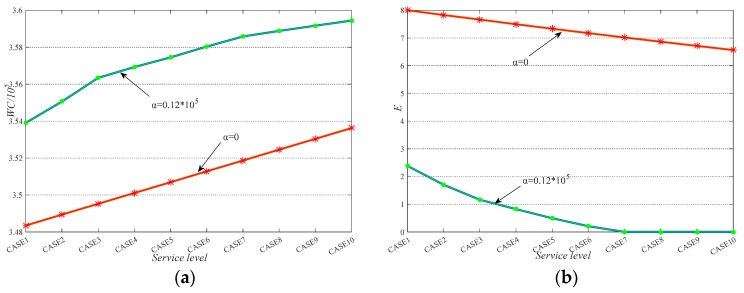
Service sensitivity analysis: (**a**) The impact of service level on WC; (**b**) the impact of service level on E.

**(III)** The results of experiment (IV) are shown in Figure 9. We see that the increase of service level leads to the increase of waiting cost. At the same time, the equity improves. In addition, the increase/decrease trend of waiting cost and equity are linear when *α* equals 0. When *α* does not equal 0, the increase/decrease rate of waiting cost and equity slows down. Moreover, from CASE7-10, we see that when the decision reaches absolute equity, the increase of service level requirement only brings the increase of waiting cost. Overall, the service level constraint is a complement of the equity objective. It not only guarantees the periodic fairness, but also increases the whole-process equity when appropriate values are set.

## 7. Conclusions

Catastrophic natural disasters cause devastating damage and leave a huge number of homeless people. An appropriate multiperiod location-allocation mechanism is necessary for the post-disaster victim resettlement process. From a perspective of humanism, we simultaneously consider victims’ waiting cost and fairness and build a DMPLA model to obtain shelter location and victim allocation planning. An illustrative example and a case study are implemented to explain the model and verify the practical applicability. 

The major conclusions of our work are as follows:

- The DMPLA model reduces people’s waiting time for resettlement through minimizing victims’ waiting costs. This improves the performance of post-disaster operations significantly from a perspective of humanism, and at the same time with an acceptable increase of monetary costs.
- The proposed DMPLA model provides an equitable decision, which is important to improve people’s satisfaction. Appropriate weight value settings can bring an obvious improvement in fairness without significantly increasing waiting costs.
- The tradeoffs between waiting cost and equity can be easily controlled by adjusting the values of weight under the support of Pareto analysis.
- Transportation capacity and building capacity significantly impact the decision. In addition, they have different binding force. Thus, it is very important to assign limited resources to purchase shelters and deliver victims in a scientific way.
- An appropriate periodic service level requirement will not lead to an obvious increase of objective value and related costs while guaranteeing the periodic equity.

Post-disaster shelter location and victim resettlement is a large-scale complex problem. Much more work needs to be done to solve practical problems. Joint optimization models considering vehicle routing, relief distribution, and evacuation simultaneously need be studied further. Besides, many other considerations can be included in the future research, such as multiple transportation modes and freight mobility planning considerations [28] in transportation planning, and training activity consideration [29,30] in evacuation planning. In addition, stochastic models considering waiting cost and fairness in post-disaster victim resettlement problems are another possible future direction.

## Figures and Tables

**Table 1:** Parameter values.

Parameter	Value
i	1, 2, 3, 4
t	1, 2, 3, 4, 5
j	1, 2, 3, 4, 5
$J^{t}$	$J^{1}:\left\{1,2\right\}$, $J^{2}:\left\{1,2,3\right\}$, $J^{3}:\left\{1,2,3,4\right\}$, , $J^{5}:\left\{1,2,3,4,5\right\}$
$A_{i}$	$A_{1}=200$, $A_{2}=400$, $A_{3}=100$, $A_{4}=300$
$B^{t}$	$B^{1}=1$, $B^{2}=2$, $B^{3}=2$, $B^{4}=3$, $B^{5}=3$
$S^{t}$(km)	$S^{1}=3,000$, $S^{2}=4,000$, $S^{3}=6,000$, $S^{4}=8,000$, $S^{5}=10,000$
$q^{t}$	$q^{1}=0.1$, $q^{2}=0.1$, $q^{3}=0.4$, $q^{4}=0.5$, $q^{5}=0.5$
C	250
α	70
γ	0.5
cb (*yuan*)	400,000
ct (*yuan*)	2

The definitions of cb and ct refer to Section 5.2, and their unit, *yuan* (RMB *yuan*), is a measure unit of currency in China. 1 *yuan* equals to about 0.158 dollar.

**Table 2:** Distance between candidate location points and demand points (km).

$d_{ij}\;(\mathrm{km})$		j				
		1	2	3	4	5
***i***	**1**	15	10	20	15	15
	**2**	8	20	40	25	8
	**3**	5	35	40	25	5
	**4**	30	50	20	18	30

**Table 3:** Experimental settings.

No.	Purposes	Experimental steps
(I)	Test the sensitivities of equity weight and analyze the tradeoffs for the waiting cost and equity	(1) Use the base setting of parameters (except *E*). (2) Generate 101 values for equity weight from 0 to 1 × 10^5^ by a step size of 0.01 × 10^5^. Solve each scenario. (3) Draw and analyze the Pareto fronts between *WC* and equity.
(II)	Test the sensitivities of periodic building capacities	(1) Use the base setting of parameters. (2) For the parameter of building capacities, adjust the value by −100% … −10%, −5% … 0 … +5%, 10% … +100%. (3) For each adjustment, consider three scenarios (equity weight equals to 0, 0.12 × 10^5^, 1 × 10^5^). Solve DMPLA.
(III)	Test the sensitivities of periodic transportation capacities	(1) Use the base setting of parameters. (2) For the parameter of periodic capacities, adjust the value by −100% … −20%, −10% … 0 … +10%, +20% … +100%. (3) For each adjustment, consider three scenarios (equity weight equals to 0, 0.12 × 10^5^, 1 × 10^5^). Solve DMPLA.
(IV)	Test the sensitivities of periodic service level	(1) Use the base setting of parameters. (2) Generate 10 sets of service level constraints by a step size of 0.5%. (3) For each adjustment, solve DMPLA.
